# Trends in Hospitalizations for Acute Kidney Injury — United States, 2000–2014

**DOI:** 10.15585/mmwr.mm6710a2

**Published:** 2018-03-16

**Authors:** Meda E. Pavkov, Jessica L. Harding, Nilka R. Burrows

**Affiliations:** 1 Division for Diabetes Translation, CDC.

Acute kidney injury is a sudden decrease in kidney function with or without kidney damage, occurring over a few hours or days. Diabetes, hypertension, and advanced age are primary risk factors for acute kidney injury. It is increasingly recognized as an in-hospital complication of sepsis, heart conditions, and surgery (*1*,*2*). Its most severe stage requires treatment with dialysis. Acute kidney injury is also associated with higher likelihood of long-term care, incidence of chronic kidney disease and hospital mortality, and health care costs (*1*,*2*). Although a number of U.S. studies have indicated an increasing incidence of dialysis-treated acute kidney injury since the late 1990s (*3*), no data are available on national trends in diabetes-related acute kidney injury. To estimate diabetes- and nondiabetes-related acute kidney injury trends, CDC analyzed 2000–2014 data from the National Inpatient Sample (NIS) (*4*) and the National Health Interview Survey (NHIS) (*5*). Age-standardized rates of acute kidney injury hospitalizations increased by 139% (from 23.1 to 55.3 per 1,000 persons) among adults with diagnosed diabetes, and by 230% (from 3.5 to 11.7 per 1,000 persons) among those without diabetes. Improving both patient and provider awareness that diabetes, hypertension, and advancing age are frequently associated with acute kidney injury might reduce its occurrence and improve management of the underlying diseases in an aging population.

Using 2000–2014 NIS data, CDC estimated the annual number of hospitalizations with acute kidney injury. NIS contains information from >7 million hospital stays from 44 states each year, estimated to represent >35 million hospitalizations nationally and >95% of the U.S. population (*4*). For this report, acute kidney injury hospitalizations were defined in two ways using the *International Classification of Diseases, Ninth Revision, Clinical Modification* (ICD-9-CM). All acute kidney injury was defined as the occurrence of at least one diagnostic code 584 (acute renal failure) or the occurrence of at least one procedure code of 39.95 (hemodialysis) or 54.98 (peritoneal dialysis). To exclude hospitalizations among patients with chronic renal failure on long-term dialysis, visits with the following procedural codes were excluded: V45.1 (renal dialysis status), V56.0 (encounter for dialysis and dialysis catheter care), V56.31 (encounter for adequacy testing for hemodialysis), V56.32 (encounter for adequacy testing for peritoneal dialysis), and V56.8 (other dialysis). Dialysis-treated acute kidney injury was defined by a diagnostic code 584 and a procedure code (39.95 or 54.98), also excluding the V-codes specified above. Hospitalizations were considered to be diabetes-related if diabetes (ICD-9-CM code 250) was listed as a diagnosis. The case definition included any hospitalization with a code for acute kidney injury regardless of cause of hospitalization.

NHIS is an annual, in-person household survey of the civilian, noninstitutionalized U.S. population that provides cross-sectional information on the health and use of health care services of the U.S. population. Data from the 2000–2014 NHIS were used to estimate the number of U.S. residents aged ≥20 years with and without diabetes. Diabetes was defined as a “yes” response to the question “Other than during pregnancy, have you ever been told by a doctor or health professional that you have diabetes or sugar diabetes?”

All acute kidney injury hospitalizations and dialysis-treated acute kidney injury hospitalizations per 1,000 persons (with and without diabetes) were calculated by dividing the estimated number of acute kidney injury hospitalizations (from NIS) by the estimated population aged ≥20 years with and without diabetes (from NHIS). Trends in all and dialysis-treated acute kidney injury were examined by sex and standardized to the 2000 U.S. standard population. Statistical software was used to obtain point estimates and standard errors based on the Taylor series linearization method and to account for complex sampling designs. Ordinary least squares regression assessed trends over time, reported as p-value for trend with two-sided significance determined as p<0.05.

The total number of hospitalizations with acute kidney injury increased from 953,926 in 2000 to 1,823,054 in 2006 and 3,959,560 in 2014 (Table). Diabetes was an associated comorbidity in 38%, 37%, and 40% of all hospitalizations in these years, respectively. During 2000–2014, the rate of all acute kidney injury hospitalizations among persons with diabetes increased by 139%, from 23.1 to 55.3 per 1,000 persons and by 230% among persons without diabetes, from 3.5 to 11.7 per 1,000 persons (both p<0.001) (Table). Similar patterns were seen for dialysis-treated acute kidney injury, but absolute rates were lower.

**TABLE:** Age-standardized rate* of hospitalization with acute kidney injury^†^ and dialysis-treated acute kidney injury^§^ among men and women aged ≥20 years with and without diagnosed diabetes, by sex and diabetes status — United States, 2000, 2006, and 2014

Characteristic	2000	2006	2014^¶^	Absolute change (95% CI)	Percent change(95% CI)
**All persons with diagnosed diabetes**					
Weighted no.	11,863,011	17,109,522	21,871,994	**—**	**—**
All acute kidney injury (no.)	364,527	666,060	1,571,265	**—**	**—**
Hospitalization rate (95% CI)	23.1 (21.5 to 24.8)	28.5 (27.0 to 29.9)	55.3 (54.1 to 56.6)	**32.2 (30.1 to 34.3)**	**139.2 (121.1 to 157.3)**
Dialysis-treated acute kidney injury (no.)	4,108	6,300	11,380	**—**	**—**
Hospitalization rate (95% CI)	0.3 (0.1 to 0.6)	0.29 (0.1 to 0.5)	0.4 (0.2 to 0.7)	**0.1 (0.0 to 0.5)**	**56.7 (-149.7 to 263.0)**
**Men with diagnosed diabetes**					
Weighted no.	5,907,203	8,203,503	10,907,239	**—**	**—**
All acute kidney injury	169,589	334,765	830,155	**—**	**—**
Hospitalization rate (95% CI)	23.0 (21.3 to 24.7)	31.5 (29.6 to 32.7)	60.9 (59.6 to 62.2)	**37.9 (35.8 to 40.0)**	**164.6 (144.6 to184.6)**
Dialysis-treated acute kidney injury (no.)	2,077	3,425	6,410	**—**	**—**
Hospitalization rate (95% CI)	0.3 (0.0 to 0.6)	0.3 (0.1 to 0.6)	0.5 (0.2 to 0.7)	**0.2 (0.0 to 0.6)**	**67.8 (-145.0 to 280.6)**
**Women with diagnosed diabetes**					
Weighted no.	5,955,808	8,906,019	10,964,755	**—**	**—**
All acute kidney injury (no.)	194,938	331,295	741,110	**—**	**—**
Hospitalization rate (95% CI)	23.2 (21.6 to 24.9)	25.8 (24.4 to 27.1)	49.7 (48.6 to 50.9)	**26.5 (24.5 to 28.5)**	**114.0 (97.8 to 130.3)**
Dialysis-treated acute kidney injury (no.)	2,031	2,875	4,970	**—**	**—**
Hospitalization rate (95% CI)	0.2 (0.0 to 0.5)	0.2 (0.02 to 0.5)	0.3 (0.1 to 0.6)	**0.1 (0.0 to 0.5)**	**43.6 (-154.8 to 242.0)**
**All persons without diagnosed diabetes**					
Weighted no.	189,675,970	202,950,590	217,677,095	**—**	**—**
All acute kidney injury (no.)	589,399	1,156,994	2,388,295	**—**	**—**
Hospitalization rate (95% CI)	3.5 (2.4 to 3.7)	6.5 (6.3 to 6.7)	11.7 (11.5 to 11.8)	**8.1 (7.9 to 8.3)**	**230.4 (216.1 to 244.7)**
Dialysis-treated acute kidney injury (no.)	8,137	12,219	16,695	**—**	**—**
Hospitalization rate (95% CI)	0.1 (0.02 to 0.1)	0.1 (0.04 to 0.1)	0.08 (0.1 to 0.1)	**0.03 (0 to 0.07)**	**64.1 (-37.4 to 165.6)**
**Men without diagnosed diabetes**					
Weighted no.	90,661,859	97,967,409	104,570,034	**—**	**—**
All acute kidney injury	316,980	617,208	1,282,955	**—**	**—**
Hospitalization rate (95% CI)	4.2 (4.1 to 4.4)	7.7 (7.5 to 8.0)	13.8 (13.6 to 14.0)	**9.6 (9.3 to 9.8)**	**225.5 (212.0 to 239.1)**
Dialysis-treated acute kidney injury (no.)	4,791	7,107	9,860	**—**	**—**
Hospitalization rate (95% CI)	0.06 (0.03 to 0.1)	0.1 (0.05 to 0.1)	0.1 (0.07 to 0.13)	**0.04 (0.0 to 0.08)**	**61.9 (-29.0 to 152.8)**
**Women without diagnosed diabetes**					
Weighted no.	99,014,111	104,983,181	113,107,061	**—**	**—**
All acute kidney injury (no.)	272,419	539,786	1,105,340	**—**	**—**
Hospitalization rate (95% CI)	2.8 (2.7 to 2.9)	5.2 (5.0 to 5.4)	9.5 (9.4 to 9.6)	**6.7 (6.5 to 6.9)**	**237.7 (222.2 to 253.2)**
Dialysis-treated acute kidney injury (no.)	3,346	5,112	6,835	**—**	**—**
Hospitalization rate (95% CI)	0.03 (0.01 to 0.1)	0.1 (0.03 to 0.07)	0.06 (0.01 to 0.08)	**0.02 (0.0 to 0.05)**	**68.0 (-52.8 to 188.8)**

**Abbreviation:** CI = confidence interval.

*Rate per 1000 population and age-standardized based on the 2000 U.S. standard population.

^†^ Acute kidney injury identified based on the following *International Classification of Diseases, Ninth Revision, Clinical Modification* (ICD-9 CM) codes: at least one diagnostic code 584 (acute renal failure) or at least one procedure code of 39.95 (hemodialysis) or 54.98 (peritoneal dialysis) and excluding the following codes: V45.1 (renal dialysis status), V56.0 (encounter for dialysis and dialysis catheter care), V56.31 (encounter for adequacy testing for hemodialysis), V56.32 (encounter for adequacy testing for peritoneal dialysis), and V56.8 (other dialysis).

^§^ Dialysis-treated acute kidney injury identified based on the following ICD-9 CM codes: at least one diagnostic code 584 (acute renal failure) and at least one procedure code of 39.95 (hemodialysis) or 54.98 (peritoneal dialysis), and excluding the following codes: V45.1 (renal dialysis status), V56.0 (encounter for dialysis and dialysis catheter care), V56.31 (encounter for adequacy testing for hemodialysis), V56.32 (encounter for adequacy testing for peritoneal dialysis), and V56.8 (other dialysis).

^¶^ All p-values for trend <0.001.

The increased rates of acute kidney injury hospitalizations affected both men and women with diabetes. Rates increased 165%, from 23.0 to 60.9 per 1,000 persons (p<0.001) among men and increased 114%, from 23.2 to 49.7 (p<0.001) among women (Figure 1) (Table). Among persons without diabetes, the rate increases were greater (226%, from 4.2 to 13.8 per 1,000 men and 238%, from 2.8 to 9.5 per 1,000 women; p<0.001); however, overall rates were substantially lower (Figure 1) (Table).

**FIGURE 1:**
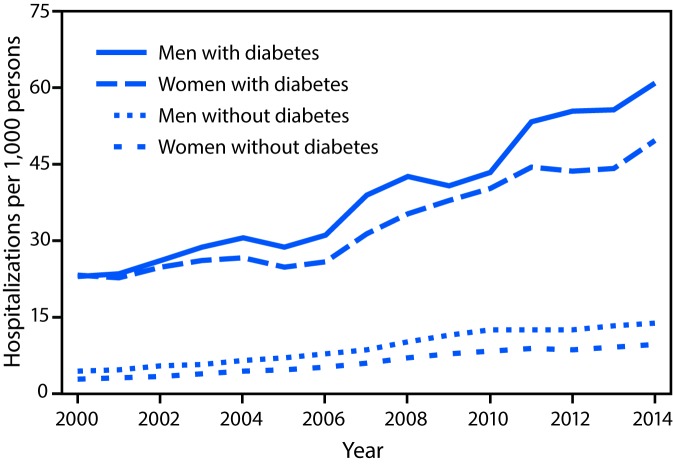
Age-standardized incidence* of hospitalizations with acute kidney injury† among men and women aged ≥20 years with and without diabetes — United States, 2000–2014 * Age-standardized based on the 2000 U.S. standard population. ^†^ Acute kidney injury identified by the following *International Classification of Diseases, Ninth Revision, Clinical Modification* codes: at least one diagnostic code of 584 or at least one procedure code of 39.95 or 54.98 and excluding the following codes: V45.1, V56.0, V56.31, V56.32, and V56.8 00–2014.

Hospitalization rates for dialysis-treated acute kidney injury increased among men and women with diabetes by 68% (from 0.3 to 0.5 per 1,000 men, p<0.001) and 44% (from 0.2 to 0.3 women, p<0.001), respectively (Figure 2) (Table). Among men and women without diabetes, the rates of dialysis-treated acute kidney injury hospitalizations were much lower, but a significant increasing trend was also observed (both p<0.001) (Figure 2) (Table).

**FIGURE 2:**
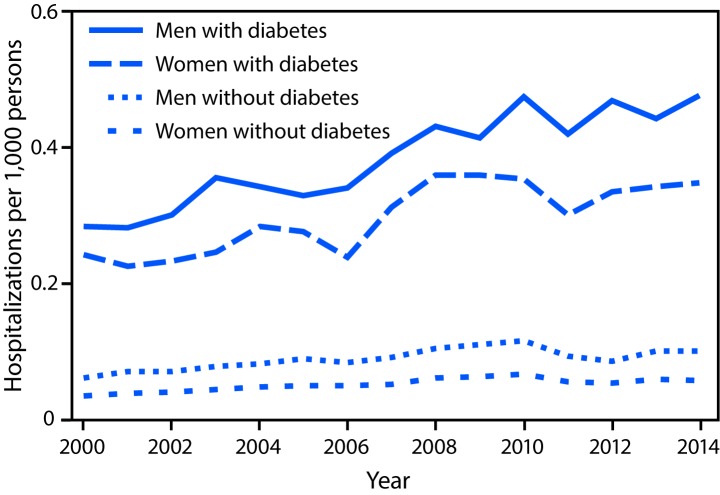
Age-standardized incidence* of hospitalizations with dialysis-treated acute kidney injury† among men and women aged ≥20 years with and without diagnosed diabetes — United States, 2000–2014 * Age-standardized based on the 2000 U.S. standard population. ^†^ Acute kidney injury identified by the following *International Classification of Diseases, Ninth Revision, Clinical Modification* codes: at least one diagnostic code of 584 and at least one procedure code of 39.95 or 54.98 and excluding the following codes: V45.1, V56.0, V56.31, V56.32, and V56.8.

## Discussion

The present analysis of nationally representative hospitalization data indicates a substantial increase in the rate of hospitalizations for acute kidney injury in men and women in the United States from 2000 to 2014, irrespective of diabetes status. Compared with persons with diabetes, acute kidney injury hospitalization rates among persons without diabetes were much lower, but the observed relative increase was larger (230% versus 139%). However, the absolute changes were much higher in persons with diabetes than in those without diabetes; persons with diabetes are nearly four times more likely to have acute kidney injury hospitalizations than are persons without diabetes. A similar absolute difference was found for dialysis-treated acute kidney injury.

The findings in this report corroborate previous reports from the United States and other countries. In the United States, unadjusted rates of first acute kidney injury hospitalization in the Medicare population with diabetes increased from 29 per 1,000 person-years in 2004 to 51 in 2014 (*2*). Among commercially insured patients aged 22–65 years with diabetes, the rate increased from 9.6 in 2005 to 15 in 2014 (*2*). Similar trends for the overall population (with and without diabetes) were reported for other large health care delivery systems such as Kaiser Permanente of Northern California (*6*). Studies in countries with national health care systems showed that dialysis-treated acute kidney injury increased more than thirteenfold in England during 1998–2013 (*7*), with the steepest increase among patients in intensive care units, and nearly threefold in Denmark during 2000–2012, particularly among elderly patients and those with multiple comorbidities (*8*). This suggests that acute kidney injury is on the rise in many counties, regardless of the health care system.

The increasing rates of acute kidney injury hospitalizations contrast with recently published data for other diabetes-related acute and chronic complications in the United States. A nationwide analysis of trends in five diabetes-related complications, including acute myocardial infarction, stroke, amputations, end-stage renal disease, and deaths from hyperglycemic crisis, indicated that rates of most complications declined during 1990–2010 (*9*). This suggests that increased survival among patients with diabetes, coinciding with a rise in other complications, such as septicemia, shock, congestive heart failure, and liver disease, might be contributing to higher rates of acute kidney injury hospitalizations (*10*).

The findings in this report are subject to at least three limitations. First, NIS data represent the number of acute kidney injury discharge diagnoses per hospital stay, not per patient. Therefore, a patient with multiple admissions during a given year might be counted several times, leading to an overestimate of the acute kidney injury incidence rate. Conversely, using administrative codes to ascertain acute kidney injury likely results in an underestimation of acute kidney injury cases caused by underrecognition and underdiagnosis. Generally, studies using change in laboratory measures, such as serum creatinine and urinary output, to define acute kidney injury provide much higher estimates of acute kidney injury incidence than those using ICD codes (*3*). Second, trends in hospitalizations with acute kidney injury codes might be influenced by changes in acute kidney injury definition (*11*), increased awareness of acute kidney injury, and changes in clinical practice over time. Data to examine these factors and their influence on hospitalizations with acute kidney injury were not available; however, the observed increases in dialysis-treated acute kidney injury might be less influenced by these factors and suggest a real increase in incidence of acute kidney injury hospitalizations over time. Finally, these data did not permit differentiation between diabetes types and diabetes duration, both of which could affect acute kidney injury hospitalizations.

Acute kidney injury increases the risk of developing or exacerbating underlying chronic kidney disease (gradual loss of kidney function over time). National health (Healthy People 2020; https://www.healthypeople.gov) objectives call for renal evaluation of patients hospitalized for acute kidney injury 6 months after discharge to monitor kidney function and prevent or delay onset of chronic kidney disease. CDC’s Chronic Kidney Disease Surveillance System monitors the prevalence of chronic kidney disease and its risk factors (including acute kidney injury) in the U.S. population and tracks progress in its prevention, management, and control.

Improving both patient and provider awareness that diabetes, hypertension, and advancing age are frequently associated with acute kidney injury is important for reversing these trends. Elderly persons have physiologically reduced kidney function and functional reserve with the appearance of global sclerosis, but also more comorbidity than do young adults, all of which heighten older persons’ susceptibility to nephrotoxic medicines, dyes used for imaging, and even dehydration, all preventable risks for acute kidney injury. Better recognition of risk factors for acute kidney injury by health care providers might improve the effectiveness of treatment of underlying conditions and prevent or mitigate additional kidney insult to patients, particularly among those hospitalized or in long-term care.

**Summary**

**What is already known about this topic?**

Clinicians increasingly recognize acute kidney injury as an in-hospital complication of sepsis, heart conditions, and surgery. It is associated with higher likelihood of long-term care, increased incidence of chronic kidney disease, increased hospital mortality, and higher health care costs. A number of U.S. studies have indicated an increasing incidence of dialysis-treated acute kidney injury since the late 1990s.

**What is added by this report?**

Analysis of data from the 2000–2014 National Inpatient Sample and the National Health Interview Surveys indicates a significant absolute and relative increase in hospitalization rates for acute kidney injury among men and women in the United States. Hospitalization for acute kidney injury among persons with diabetes accounted for approximately 40% of all such hospitalizations; absolute increases in hospitalization rates among persons with diabetes were larger than those among persons without diabetes.

**What are the implications for public health and health care practice?**

Diabetes is a known risk factor for acute kidney injury. The increasing number of persons living with diabetes is likely to also increase the number of persons with acute kidney injury. Improved awareness by health care providers that diabetes, hypertension, and advanced age are important risk factors for acute kidney injury might reduce its occurrence and improve management of the underlying diseases in an aging population.
